# Monitoring storage induced changes in the platelet proteome employing label free quantitative mass spectrometry

**DOI:** 10.1038/s41598-017-11643-w

**Published:** 2017-09-08

**Authors:** Maaike Rijkers, Bart L. van den Eshof, Pieter F. van der Meer, Floris P. J. van Alphen, Dirk de Korte, Frank W. G. Leebeek, Alexander B. Meijer, Jan Voorberg, A. J. Gerard Jansen

**Affiliations:** 1 Department of Plasma Proteins, Sanquin-AMC Landsteiner Laboratory, Amsterdam, The Netherlands; 2 Department of Product and Process Development, Sanquin Blood Bank, Amsterdam, The Netherlands; 3 Department of Research Facilities, Sanquin, Amsterdam, The Netherlands; 4 Department of Blood Cell Research, Sanquin-AMC Landsteiner Laboratory, Amsterdam, The Netherlands; 5 Department of Hematology, Erasmus University Medical Centre, Rotterdam, The Netherlands; 6 Department of Pharmaceutics, Utrecht Institute for Pharmaceutical Sciences (UIPS), Utrecht University, Utrecht, The Netherlands; 7 Department of Vascular Medicine, Amsterdam Medical Centre, Amsterdam, The Netherlands

## Abstract

Shelf life of platelet concentrates is limited to 5–7 days due to loss of platelet function during storage, commonly referred to as the platelet storage lesion (PSL). To get more insight into the development of the PSL, we used label free quantitative mass spectrometry to identify changes in the platelet proteome during storage. In total 2501 proteins were accurately quantified in 3 biological replicates on at least 1 of the 7 different time-points analyzed. Significant changes in levels of 21 proteins were observed over time. Gene ontology enrichment analysis of these proteins revealed that the majority of this set was involved in platelet degranulation, secretion and regulated exocytosis. Twelve of these proteins have been shown to reside in α-granules. Upon prolonged storage (13–16 days) elevated levels of α-2-macroglobulin, glycogenin and Ig μ chain C region were identified. Taken together this study identifies novel markers for monitoring of the PSL that may potentially also be used for the detection of “young” and “old” platelets in the circulation.

## Introduction

Platelet transfusion is commonly used to restore platelet count and prevent bleeding in thrombocytopenic patients and patients with platelet dysfunctionality. The shelf life of platelet concentrates (PCs) is limited to a maximum storage time of 5–7 days, depending on national guidelines for platelet transfusion. Prolonged platelet storage leads to a decrease in platelet functionality known as the platelet storage lesion (PSL)^1, 2^. During storage, platelet morphology changes and the discoid shape is lost^2^. Furthermore, platelets become activated, as is evidenced by surface exposure of P-selectin, which is a consequence of α-granule release. PI3-kinase dependent Rap1 activation, which leads to activation of integrin αIIbβ3, has been implicated in the development of the PSL^3^. In addition, levels of membrane proteins such as GPIbα and GPV decline during storage^1, 4, 5^. These proteins are cleaved by ADAM17, which is dependent on p38 mitogen-activated protein kinase signaling^6^. Functionality of platelets is reduced upon storage, as has been shown by a decreased response to agonists like ADP, collagen, ristocetin and PAR1 activating peptides^1, 2, 4^. During storage, changes in platelet metabolism occur^7^. Also an increase in levels of reactive oxygen species leading to oxidative stress has been reported^8^. Despite these findings, triggers for initiating the development of the PLS are still not fully elucidated. Recent improvements in storage methods can increase platelet quality, and new platelet additive solutions seem to prolong platelet function^9, 10^. However, the *in vitro* hallmarks of the PSL are still observed after longer storage periods^4^.

Several proteomic studies on stored platelets have been performed^3, 11–13^. Supernatant of stored platelets^14–18^, platelet shedding^19^ and platelet releasates^20^ have been analyzed by mass spectrometry. Employing 2D gel/differential in gel electrophoresis (2D/DIGE), decreased levels of septin-2, β-actin and gelsolin^11^ and increased levels of Rap1^3^ were found during platelet storage. Using different mass spectrometry approaches (2D gel/DIGE, iTRAQ and ICAT) Thon *et al*. showed that fibrinogen was consistently downregulated upon storage. The total number of proteins identified in this study ranged from 93 (2D/DIGE) to 355 proteins (iTRAQ)^12^. The proteome of non-stored platelets has been shown to consist of at least 4200 proteins^21–24^.

In previous studies on platelet storage, only limited numbers of proteins were identified. Label free quantitative (LFQ) mass spectrometry has been successfully used to obtain large quantitative data sets^25^. Here we used LFQ mass spectrometry to monitor changes in the platelet proteome during storage. In contrast to previous studies we also monitored changes in the platelet proteome during extended storage since this may help to dissect pathways involved in development of the PSL.

## Results

### General results from LFQ mass spectrometry analysis

**Figure 1:**
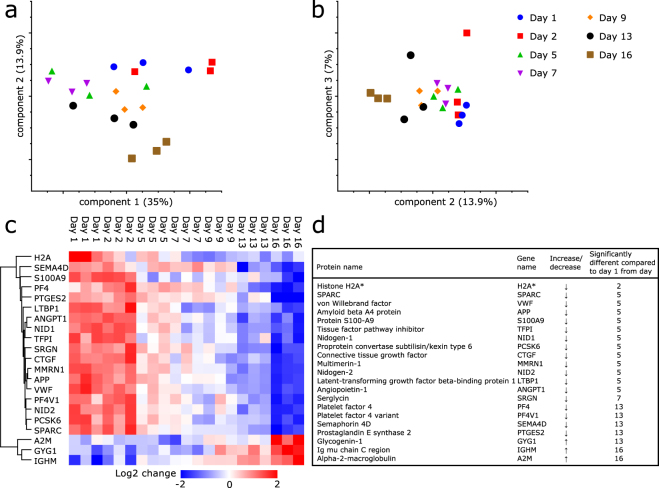
Principle component analysis and proteins with significantly changed levels during platelet storage. (**a**) Principle component analysis performed on proteins identified in all samples measured (n = 1753). Principle component (PC) 1 captures 35%, PC2 13.9%. (**b**) Including PC3, 7% of differences were explained. (**c**) Heat map and hierarchical clustering (based on average Euclidean distance and preprocessed with k-means) of proteins with significantly changed levels (n = 21). Proteins are indicated by gene names. Heat map colors (see legend) are based on the z-scored LFQ data (log2) reported in Supplementary Table S1. (**d**) List of 21 proteins changing in abundancy during storage. Time period from which protein was significantly different compared to day 1 is indicated (P < 0.05). *Peptides identified and quantified for H2A correspond to the sequence of more than 1 histone H2A variant. These peptides originate from one or more of the following histones: H2AFJ, HIST1H2AJ, HIST1H2AH, HIST1H2AC, HIST3H2A, HIST1H2AD, HIST1H2AG, HIST1H2AB, HIST2H2AB and H2AFX.

Platelets were stored in plasma under standard blood bank conditions. Functional properties of platelets during storage under these conditions have been described previously^4^. To monitor changes in the composition of the platelet proteome during storage, samples were collected at different time points. Employing LFQ mass spectrometry a total of 3451 proteins was identified. A principle component analysis (PCA) was performed to compare the changes in the platelet proteome induced by storage to the data corresponding to the total number of proteins identified in all samples (Fig. 1a,b). Generally, samples from the same storage day cluster together; however not all time points are clearly separated. The 3 replicates of day 16 are most separate from other samples, day 1 and 2 are in close proximity and day 5 and 7 cluster together. The relative limited clustering of biological replicates implicates that storage induces only small differences in protein abundances. For further analysis proteins that were not reproducibly found in 3 biological replicates corresponding to at least 1 time point were excluded. This resulted in a dataset which contained 2501 proteins. Levels of 21 out of the 2501 accurately quantified proteins changed significantly during the 16 days of storage (Fig. 1c,d and Supplementary Data S1).

Of the 21 significantly changed proteins, 18 decreased in abundancy over time, and 3 proteins: α-2-macroglobulin (A2M), Ig μ chain C region (IGHM) and glycogenin-1 (GYG1), were up-regulated. To determine from which time point on the protein levels were significantly changed compared to day 1, a post-hoc test (Dunnett’s multiple comparisons test) was performed for all 21 proteins independently (Fig. D). Histone H2A (H2A) was significantly decreased at day 2 compared to day 1. A set of 12 proteins was significantly reduced from day 5 onwards, mainly consisting of proteins known to reside in α-granule proteins like VWF and amyloid beta A4 protein (APP). The 3 proteins displaying increased levels were significantly different from day 13 (GYG1) and day 16 (IGHM and A2M). In general, the changes in protein levels, as described in LFQ values, were relatively small compared to the biological variation between the 3 replicates, indicating that during storage, changes in levels of individual proteins are limited (all log2 LFQ data can be found in Supplementary Table S1).

### GO-term enrichment and STRING analysis

**Figure 2:**
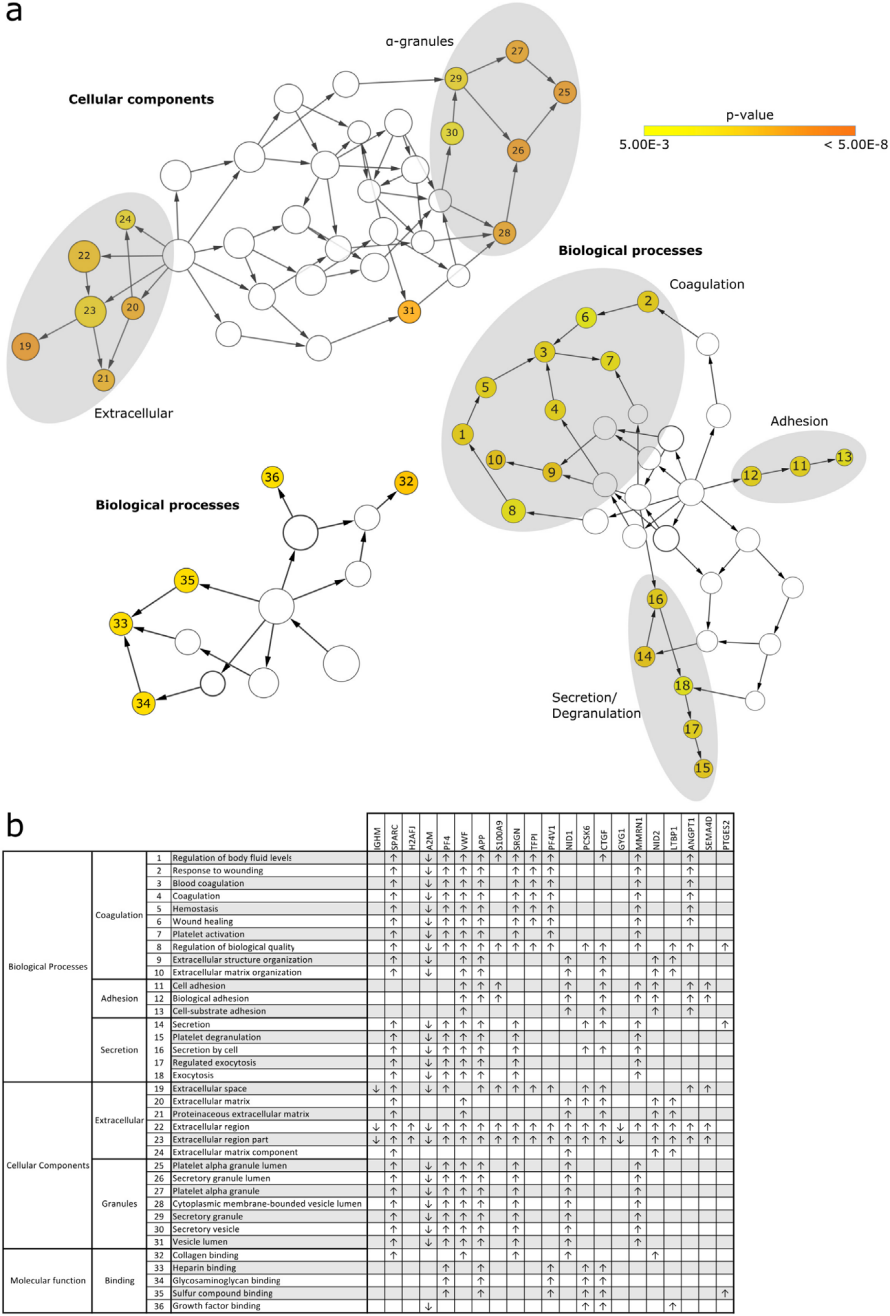
GO term enrichment analysis of cellular components, biological processes and molecular functions. (**a**) Each circle represents a GO term for which the numbers are specified in the table. GO terms are significantly enriched if P < 0.005 and are colored according to their P value (see Panel A). (**b**) Each row represents a significantly enriched GO term and each column shows one of proteins with significantly changed levels indicated by their gene name. An ‘x’ indicates that the GO term has been assigned to the protein.

To get a more general overview of changes during storage and analyze which processes are most affected during storage, GO term enrichment analysis was performed. The 21 proteins with significantly changed levels during storage were compared to all accurately quantified proteins (n = 2501) and analyzed for enriched GO terms using the BiNGO plug-in in CytoScape (P < 0.005, correction for multiple testing using Benjamini&Hochberg (FDR) correction). GO terms^26^ linked to biological processes, cellular component and molecular function were analyzed. Three different clusters of enriched biological processes were found: adhesion, secretion and coagulation (Fig. 2). Analysis of the cellular localization of the changed proteins revealed that a large portion of these proteins are localized in α-granules. This indicates that the release of α-granules is an early event during platelet storage. Besides, proteins with the GO annotation “extracellular space” were enriched. Many proteins belong to both of these groups, as these proteins are secreted upon degranulation and then end up in the extracellular space (Fig. 2). Molecular functions for which the group of changed proteins are enriched are collagen binding, heparin binding, glycosaminoglycan binding, sulfur compound binding and growth factor binding.

**Figure 3:**
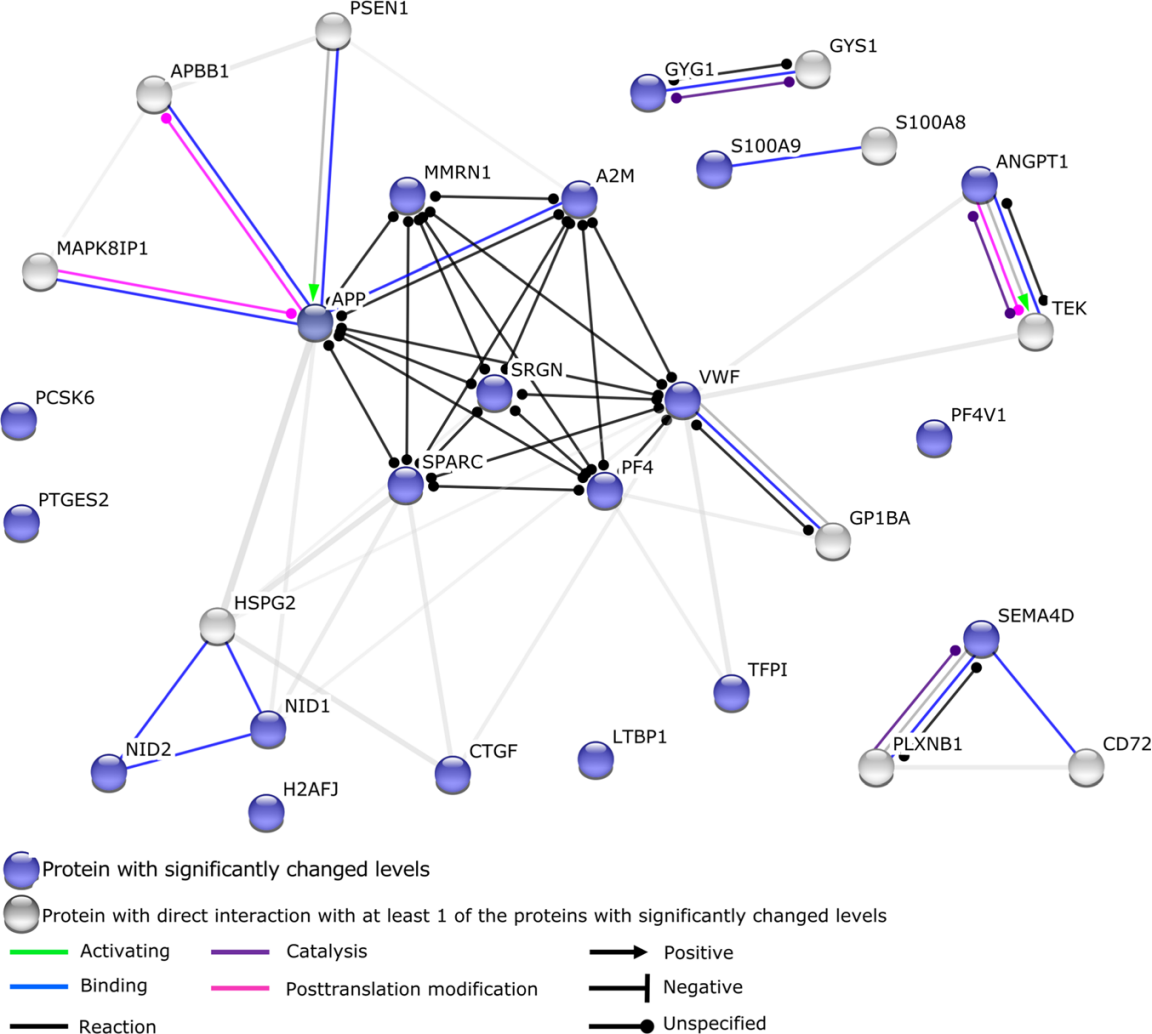
STRING analysis. STRING analysis monitors potential interaction between the candidate proteins identified in this study. All proteins with significantly changed levels during storage were included, except for IGHM (constant region of heavy chain IgM) which is not annotated in the STRING analysis tool. Blue colored circles represent proteins levels of which changed significantly during storage. Grey circles represent proteins which directly interact with at least one of the proteins of which levels were changed significantly during storage. Interactions between different proteins are indicated by colored lines and arrowheads as depicted in the figure.

STRING analysis was employed to monitor potential interaction between the candidate proteins identified in this study. The main interactive cluster consists of MMRN1, APP, SPARC, SRGN, PF4, VWF and A2M, which is consistent with the presence of these components in α-granules (Fig. 3). Via so-called textmining (an algorithm included in the STRING analysis that selects proteins that are frequently mentioned together in the scientific literature) TFPI, ANGPT1, NID1, NID2 and CTGF are also connected to this cluster. For H2A, LTBP1, PTGES2, PCSK6 and PF4V1 no binding partners were identified. STRING analysis identified CD72 and PLXNB1 as putative binding partners for SEMA4D; CD72 and PLXNB1 were however not identified in our current analysis of the platelet proteome. GYS1, a binding partners of GYG1 was identified in platelets but levels of GYS1 did not change during storage. S100A8 was identified as a binding partner of S100A9; levels of S100A8 declined over time, however these changes were not significant.

### Alpha-granule proteins

**Figure 4:**
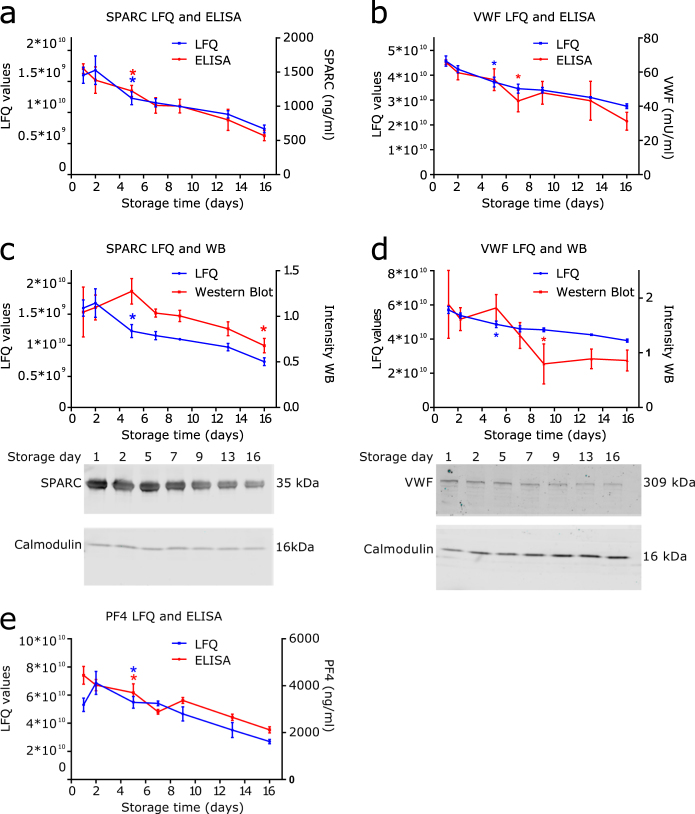
Reduced levels of α-granule proteins SPARC, VWF and PF4 during storage. (**a**) Non-imputed LFQ values of SPARC (blue) and SPARC levels as measured with ELISA (red). (**b**) Non-imputed LFQ values of VWF (blue) and VWF levels as measured with ELISA (red). (**c**) Western blot analysis of SPARC, LFQ values (blue) and intensities of Western blot (red). (**d**) Western blot analysis of VWF, LFQ values (blue) and intensities of Western blot (red). Calmodulin was used as loading control. Bands were normalized according to intensity of calmodulin (loading control), and average intensity per membrane was normalized to 1. (**e**) Non-imputed LFQ values of PF4 (blue) and PF4 levels as measured with ELISA (red). Data represents mean ± standard deviation (n = 3), *P < 0.05 compared to day 1 from this time point onwards. Uncropped immunoblots of SPARC, VWF and calmodulin can be found in Supplementary Data S5.

Among the 18 proteins which were less abundant at the end of the storage period compared to the fresh platelets, 7 (SPARC, VWF, APP, NID1, MMRN1, SRGN and PF4) have the GO term “platelet α-granule” but even 5 more (PCSK6, CTGF, LTBP1, ANGPT1 and PF4V1) have been reported to be present in α-granules^27, 28^. The data suggests a gradual release of α-granules as 9 of the 12 decreased α-granule proteins are significantly decreased from day 5 onwards (SRGN from day 9 and PF4 and PF4V1 from day 13) (Fig. 2b). Analysis of SPARC, VWF and PF4 levels by ELISA correlates with the proteomic quantification of these proteins (Fig. 4a,b,e). Also Western blot analysis confirmed a decrease of these proteins during platelet storage (Fig. 4c,d).

### Dense granules and lysosomes during storage

**Figure 5:**
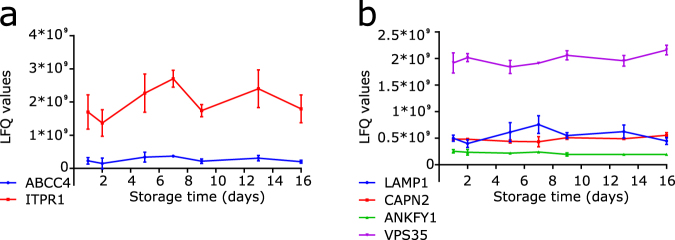
Levels of dense granule and lysosomal proteins during storage. (**a**) Non-imputed LFQ values of dense granule proteins ABCC4 (blue) and ITPR1 (red). (**b**) Non-imputed LFQ values of lysosomal proteins LAMP1 (blue), CAPN2 (red), ANKFY1 (green) and VPS35 (purple). Levels of these proteins do not significantly change during storage. Data represents mean ± standard deviation (n = 3), *P < 0.05 compared to day 1 from this time point onwards.

Besides α-granule release, platelet activation can lead to the release of dense granules and lysosomes^29^. In an earlier study, we observed a reduced platelet aggregation response to ADP upon storage^4^. This is caused by desensitization of the P2Y1/P2Y12 receptors induced by leakage of ADP from platelets during storage due to dense granule release^30^. Dense granules only contain a limited number of proteins, among these are multidrug resistance-associated protein 4 (ABCC4) and inositol 1,4,5-trisphosphate receptor type 1 (ITPR1) (Fig. 5a). According to our data there is no evidence for the loss of these dense granule proteins during storage. However, as these proteins are present on the membrane of dense granules, fusion of dense granule membrane with the platelet membrane might lead to exposure of these proteins on the outside of the platelet rather than loss of these proteins.

Known platelet lysosomal markers are LAMP-1 and CD63 (or LAMP-3)^29, 31^. CD63 was not detected in the mass spectrometry analysis. LAMP1 was detected, and shows no significant differences during storage. Other proteins which have been reported in lysosomes (calpain 2 (CAPN2)^32^, ankyrin repeat and FYVE domain-containing protein 1 (ANKFY1)^33^ and vacuolar protein sorting-associated protein 35 (VPS35)^33^) remain at a similar level (Fig. 5b), suggesting limited loss of lysosomes during platelet storage.

### Non-alpha granule proteins reduced during storage

**Figure 6:**
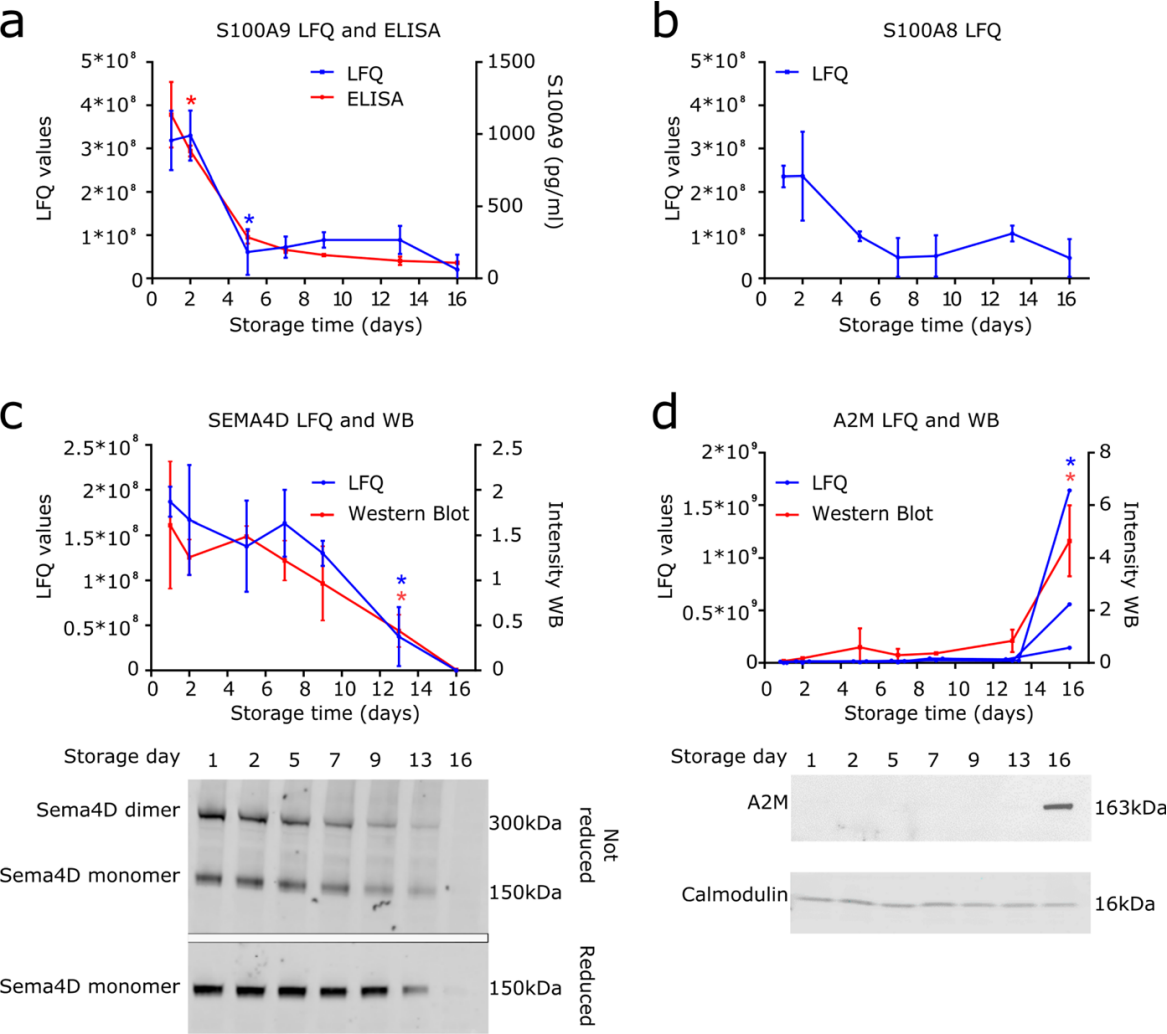
Non-α-granule proteins with reducing levels during storage. (**a**) Non-imputed LFQ values and concentrations measured employing ELISA of S100A9. (**b**) Non-imputed LFQ values of S100A8. (**c**) Non-imputed LFQ values and Western blot analysis of SEMA4D including a representative Western blot analysis of SEMA4D under reducing and non-reducing conditions. (**d**) Non-imputed LFQ values and Western blot analysis of A2M including a representative Western blot of A2M. Calmodulin was used as loading control. Band normalization was performed as in Fig. 4. Data represents mean ± standard deviation (n = 3), in (**d**) values of all replicates are shown for LFQ values. *P < 0.05 compared to day 1 from this time point onwards. LFQ values of 0 indicates that protein was not detected. Uncropped immunoblots of SEMA4D, A2M and calmodulin can be found in Supplementary Data S5.

From the 18 down-regulated proteins during storage, 12 have been described to reside in α-granules, the remaining 6 proteins are localized in distinct subcellular compartments. Interestingly, levels of two peptides assigned to H2AJ (but also present in other H2A histones: see Supplementary Data S2) were significantly lower from day 2 onwards; these H2A derived peptides were detected until day 5 (and in 1 sample on day 7). The role of histones in platelets is unclear, as platelets only contain mitochondrial DNA which does contain DNA binding proteins but no histones^34^. Confocal microscopy employing a polyclonal antibody directed towards the first 30 amino acids of H2AFJ revealed that H2AFJ (or other member of the H2A histone family are indeed present in platelets (Supplementary Data S2). Protein S100-A9 (S100A9), also known as MRP14, was significantly decreased from day 5 onwards. Proteomic quantification of S100A9 was confirmed employing ELISA (Fig. 6a). S100A9 appears as a heterodimer with protein S100-A8 (S100A8)^35^, also known as MRP8, and together with CD36 plays a role in thrombosis^36, 37^. Patients with acute myocardial infarction have elevated levels of S100A9^36^ and it was shown in mice that absence of S100A9 leads to prolonged time to arterial occlusion^37^. Although changes in S100A8 were not significant according to the analysis performed, a similar decline of levels of S100A8 was observed as for S100A9 (Fig. 6b). LFQ values of semaphorin 4D (SEMA4D) were significantly reduced from day 13 onwards. It has been described previously that in platelets activated by agonists, SEMA4D is shed by ADAM17^38^. In our dataset, ADAM17 was not identified; this is probably due to the low levels of ADAM17 in platelets. Using Western blot analysis we could confirm the loss of SEMA4D during storage. As SEMA4D occurs as homodimers^39^, Western blot analysis was performed under reducing and non-reducing conditions. A decline in the levels of both the homodimer as well as individual Sema4D subunits was observed (Fig. 6c). We also found the non-α-granule proteins tissue factor pathway inhibitor (TFPI), nidogen-2 (NID2) and prostaglandin E synthase 2 (PTGES2) to be significantly reduced upon platelet storage (Fig. 1 and Supplementary Data S1).

The possibility of leukocyte contamination was ruled out by comparing our data set with previously published data on protein abundancy in neutrophils and monocytes^40^, contaminants in murine platelet lysates^38^, RNA-seq data of platelets^41^ and megakaryocytes^42^ (Supplementary Data S3). For preparation of PCs, leukocyte reduction filters were employed (see Material and Methods). This strongly reduces the amount of leukocytes to <10.000 per 3.4 × 10^11^ platelets. We monitored the effect of leukocyte filtration on S100A9 content of platelet lysates (Supplementary Data S4). Two consecutive leukocyte filtration steps dramatically reduced the leukocyte content but had no effect on the S100A9 content of PC (Supplementary Data S4). The presence of H2A in platelets was confirmed employing confocal microscopy (Supplementary Data S2). These analyses suggest that proteins with significantly changed levels during storage all originate from platelets.

### Binding and/or endocytosis of proteins by platelets during storage

A slow but gradual increase of IGHM was observed in platelets during storage, however, compared to day 1 the increase was only significant at day 16. IGHM was detected in all samples analyzed on all time points, indicating that, although only in small amounts, IGHM was already present in fresh platelets. GYG1, a glycogenin glucosyltransferase involved in glycogen synthesis, also increased during storage, which was significant from day 13 onwards. The third protein which increased during storage was A2M, a proteinase inhibitor which is present in plasma but has also been found in platelet α-granules. A2M was not detected by the mass spectrometer until day 9 and is only significantly increased at day 16 of storage, as was confirmed by Western blot (Fig. 6d).

## Discussion

Employing LFQ mass spectrometry we provide a data set of 2501 accurately quantified proteins that are present in stored platelets. Levels of a subset of proteins declined significantly over time; the majority of these proteins localize in α-granules suggesting that release of this storage compartment is an early event during platelet storage. *In vivo*, proteins released from α-granules have been shown to contribute to coagulation, inflammation and angiogenesis^43^. Reduced numbers of α-granules in stored platelets might thus influence the *in vivo* function of transfused platelets. In our study we only detect a decline in a subset of proteins that have been reported to reside in α-granules. Some known α-granule proteins were not identified (like CCL2 and MMP1)^43^, other α-granule proteins such as coagulation factor V and C-C motif chemokine 5 were identified and quantified but displayed no significant differences over time. Proteins residing in α-granule membranes remain associated to the platelet membrane following fusion of α-granules with the plasma membrane. Therefore overall levels of these transmembrane proteins are not expected to decline during storage. This provides an explanation for the lack in decline in levels of transmembrane proteins like P-selectin. Besides α-granules, platelets contain dense granules and lysosomes, which upon activation can be secreted. For the dense granule proteins multidrug resistance-associated protein 4 and inositol 1,4,5-trisphosphate receptor type 1, no significant changes were found over time. Also lysosomal proteins LAMP1, calpain 2, ankyrin repeat and FYVE domain-containing protein 1 and vacuolar protein sorting-associated protein 35 did not change in abundancy during platelet storage. On the contrary, we and others showed desensitization of the ADP receptors P2Y1 and P2Y12, caused by continuous leakage of dense granules during storage^30, 44^. This apparent discrepancy can be explained by translocation of dense granule and lysosomal components to the plasma membrane instead of their release from platelets. Our dataset does not contain proteins present in the lumen of dense granules and lysosomes. Therefore, the data obtained in this study do not allow for monitoring of the contribution of dense granule and lysosomal release to the PSL.

Prudova *et al*. used terminal amine isotope labeling of substrates (TAILS) to study proteolysis of platelet proteins during storage. Their data showed extensive metalloproteinases mediated processing of platelet proteins which included GPIbα, GPV and GPVI during storage^13^. In our study we did not identify significant changes in levels of GPIbα and GPV. Also in a previous study we did not observe significant changes in surface expression of GPIbα and GPV until day 9 of storage^4^. Our LFQ based mass spectrometry approach allows for monitoring of global changes in protein levels but does not allow for robust quantification of individual peptides. Therefore small amounts of proteolytically processed platelet surface receptors like GPIbα and GPV cannot be accurately measured. Using iTRAQ, Thon *et al*. showed that levels of 299 out of 335 proteins changed during storage^12^. These proteins included SPARC, VWF, NID1, NID2, LTBP1, PF4, PF4V1 and MMRN1 that were also found in our study (Fig. 1). We only found differences in protein levels during storage for a limited number of proteins when compared to Thon and coworkers^12^. Thirteen proteins we identified have, as far as we know, not been implicated previously in development of the PSL (H2A, APP, TFPI, ANGPT1, SRGN, SEMA4D, PTGES2, S100A9, PCSK6, CTGF, GYG1, IGHM and A2M). Differences between our study and previous studies are most likely caused by the different methods employed for protein identification and quantification. Thiele *et al*.^11^ and Schubert *et al*.^3^ used 2D/DIGE methods to identify proteins changing in abundancy during platelets storage. A decline in levels of septin-2, β-actin and gelsolin^11^, and an increase of rap1^3^ have been linked to the PSL. In our study we did detect these proteins, but did not find significant changes in their levels upon storage. Studies focusing on proteins released in the supernatant employing either 2D/DIGE methods^14, 15^ or an antibody based microarray^16^ during platelet storage identified α-granule proteins (vitronectin, clusterin, integrin-linked kinase and TLT-1) and several cytokines including CCL5 and CXCL7 to be released from the platelets during storage. Employing quantitative label free plasma QconCAT based targeted proteomics, Dzieciatkowska and co-workers monitored levels of a panel of 109 proteins present in the supernatant of stored platelets^18^. Also their data revealed changes in the proteome of platelets upon storage which were more pronounced in female donors when compared to male donors^17, 18^. Our results show considerable overlap with the protein content of platelet releasates identified in previous studies^45–47^. These mainly include α-granule proteins like VWF, SPARC, NID1, MMRN1 and PF4^45–47^ but also some other proteins like IGHM^45, 47^, confirming its presence in platelets. It has been shown that besides α-granule proteins, also proteins like kininogen-1, serpin proteinase inhibitor 8 and beta tropomyosin are released from platelets during storage, suggesting progressive leakage or degranulation^14–16^. Also in our study we observed release of non-α-granule proteins such as SEMA4D (Fig. 6). These studies are consistent with our observation that α-granule release is as an early event during storage and also show that non-α-granule proteins can be secreted from platelets during storage.

As expected we observed a decline in levels of a subset of proteins during storage. However, upon prolonged storage we also observed elevated levels of A2M, IGHM and GYG1. This suggests that platelets ingest or bind these proteins during storage. Since we only observe this phenomenon upon prolonged storage (after 13 and 16 days), the relevance of this observation remains to be established. Strikingly, H2A was identified in platelets, while histones are not expected in anucleated cells such as platelets. Small amounts of leukocytes in PCs could have accounted for the identification of H2A at early time points of storage. However, confocal microscopy employing an anti-H2AFJ antibody confirmed the presence of H2A histone family members in platelets (Supplementary Data S2). In agreement with our findings, H2A histone family members were classified as a true part of the platelet proteome^38^. Additionally, H2A histone family members have been detected in platelet microparticles^48^.

Leukodepletion employing filtration is used as a standard procedure to reduce the number of leukocytes in PCs^4^. The maximum number of leukocytes in PCs containing 3.4 × 10^11^ platelets is below 10^4^ (Supplementary Data S4). Contaminating leukocytes consist primarily of lymphocytes (90%) and it has been shown that there are only 0–100 granulocytes per PC (de Korte *et al*., unpublished observations). To further exclude that the identified S100A9 originates from neutrophils we analyzed S100A9 levels in non-leukodepleted and singly and doubly leukodepleted PCs (Supplementary Data S4). Leukodepletion did not have impact on S100A9 levels in PCs showing that S100A9 is a bona fide component of platelets. S100A9 has been identified both in platelets and megakaryocytes^21, 41, 49^. Platelet derived S100A9 has been shown to regulate thrombosis^37^. Our findings therefore identify S100A9 as a potential marker for development of the PSL.

In conclusion, we identified a total of 2501 proteins of which 21 proteins were significantly changed during platelet storage. It is interesting that although platelet functionality significantly decreases, especially after prolonged storage of more than 7 days^4^, changes on protein level appear to be relatively minor. This suggests that the overall protein composition of platelets is only limitedly affected whereas their ability to respond in a coordinated manner to agonists is greatly reduced. Although the intra-donor variation was minimized by using PCs composed of pools of 22 donors per PC, the observed variation between the three biological replicates may mask subtle changes in the proteome of stored platelets. Currently, no platelet proteins have been assigned as markers for either “young” or “old” platelets. If indeed present in platelets, H2A histone family members may provide a marker for “young” platelets; also expression of protein S100A9, which rapidly declines at day 5 of storage may be confined to young platelets. Similarly, A2M, IGM and GYG1 may be suitable as a biomarker for “aged” platelets. This should be confirmed by future studies.

## Materials and Methods

### Platelet concentrate preparation and storage

Blood was drawn from healthy, anonymized volunteers in accordance with Dutch regulations and after approval from the Sanquin Ethical Advisory Board in accordance with the Declaration of Helsinki. Written informed consent was obtained from all participants.

Buffy coats (BCs) were obtained from whole blood as described before^50^. For each platelet concentrate (PC) a pool of 22 buffy coats was used. PCs were prepared essentially as described previously, following addition of plasma and centrifugation, the platelet-rich supernatant was transferred the storage container through a leucoreduction filter (from a TF*FP0610M1 pooling system (Terumo, Tokyo, Japan))^4, 50^. This protocol corresponds to the standard procedure for the production of PCs at Sanquin Blood Bank. PCs were stored in plasma under standard blood bank conditions in a temperature-controlled cabinet (Helmer Scientific, Noblesville, IN, USA) at 20–24 °C on a flatbed at 60 strokes per minute, as described in detail previously^4^. In each of the units a sample site coupler was inserted and aseptic sampling was performed with a syringe at day 1, 2, 5, 7, 9, 13 and 16. To avoid red blood cell contamination as much as possible, platelets were spun down for 20 min at 120 *g*, then 10% (v/v) ACD (85 mM trisodium citrate, 71 mM citric acid, 111 mM glucose) was added to the platelets in plasma, followed by 3 washes in platelet wash buffer (PWB) (containing 36 mM citric acid, 103 mM NaCl, 5 mM KCl, 5 mM EDTA, 5.6 mM glucose, pH 6.5). Washed platelets were resuspended in PWB containing 10% (v/v) ACD and stored at −20 °C.

### Sample preparation for LFQ Mass Spectrometry

About 100 × 10^6^ platelets in 25 µl wash buffer were lysed in 8 M urea in 100 mM Tris-HCl (pH 8). The protein concentration was determined and 5 μg of protein was used for sample preparation for mass spectrometry. Disulphide bonds were reduced with 10 mM DTT for 60 minutes at 20 °C, alkylated with 55 mM iodocetamide for 45 minutes at 20 °C, and samples were digested overnight at 20 °C with MS-grade trypsin (Promega, Medison, WI, USA) (in a protein:trypsin ratio of 20:1). Peptides were desalted and concentrated using Empore-C18 StageTips^51^ and eluted with 0.5% (v/v) acetic acid, 80% (v/v) acetonitrile as described before^52^. Sample volume was reduced by SpeedVac and supplemented with 2% (v/v) acetonitrile, 0.1% (v/v) TFA to a final volume of 5 μl. Three μl was injected in the mass spectrometer (Orbitrap Fusion, Thermo Scientific, Waltham, MA, USA).

### Mass spectrometry analysis

Tryptic peptides derived from were separated by nanoscale C18 reverse chromatography coupled on line to an Orbitrap Fusion Tribrid mass spectrometer (Thermo Scientific) via a nanoelectrospray ion source (Nanospray Flex Ion Source, Thermo Scientific), using the same settings as described by Gazendam *et al*.^52^. All MS data were acquired with Xcalibur software (Thermo Scientific).

### Mass spectrometry data analysis

The RAW mass spectrometry files were processed with the MaxQuant computational platform, 1.5.2.8^53^. Proteins and peptides were identified using the Andromeda search engine by querying the human Uniprot database (downloaded February 2015)^33^. Standard settings with the additional options match between runs, LFQ, and unique peptides for quantification were selected. The generated ‘proteingroups.txt’ table was filtered for reverse hits, ‘only identified by site’ and potential contaminants using Perseus 1.5.1.6. The LFQ values were transformed in log2 scale.

Samples were grouped per time point (7 groups, 3 samples per group) and proteins were filtered for at least 3 valid values on at least one of the 7 time points. Missing values were imputed by normal distribution according to default settings in Perseus (width = 0.3, shift = 1.8)^54^, assuming these proteins were close to the detection limit. The global changes in protein levels were assessed employing the analysis-of-variance function of Perseus, proteins with FDR p-values lower than 0.05 (S0: 0.2) were considered to be significantly changed over time. A post-hoc test (Dunnett’s multiple comparisons test) was performed to determine from which time point on significant changes in protein levels were observed when compared to day 1 of storage. Principle component analysis (PCA) was performed using the total number of proteins identified in all samples (in 3 biological replicates on 7 time points).

Gene ontology enrichment analysis of biological processes, molecular functions and cellular compartments of the significantly different proteins was performed using the CytoScape (version 3.3.0)^55^ plug-in BiNGO (version 3.0.3)^56^. Ontology and annotation datasets were downloaded on April 4, 2016 from the Gene Ontology Consortium website (www.geneontology.org). The significantly differentially expressed proteins were compared to the accurately quantified proteins in the dataset. GO terms were assigned significantly enriched when p < 0.005. STRING analysis was performed on the proteins of which the abundancy changed significantly over time, employing the online application on string-db.org^57^.

### Immunoblotting

Washed platelets were lysed in lysis buffer (0.5% (v/v) NP40, 10% (v/v) glycerol, 50 mM Tris-HCl, 100 mM NaCl, 1 mM EDTA, pH 7.4 and complete protease inhibitor cocktail (Roche Diagnostics, Mannheim, Germany), 1 tablet per 50 mL) for 1 h on ice followed by 10 minutes centrifugation at 40817 *g* to obtain the lysed proteins. Sample buffer (62.5 mM Tris-HCl, 2% (v/v) SDS, 10% (v/v) glycerol, 0.01% (v/v) bromophenol blue, pH 6.8) and 10 mM DTT was added to the lysates, the samples were separated on SDS PAGE gels (NuPAGE® Novex® 4–12% Bis-Tris, Thermo Scientific) and proteins were blotted on nitrocellulose membranes using the iBlot® 7-Minute Blotting System (Thermo Scientific). The membranes were blocked with Odyssey® Blocking Buffer (PBS) (LI-COR Biotechnology, Lincoln, NE, USA) for 30 min, washed 3 times in PBS, 0.1% (v/v) Tween20 and incubated with the appropriate antibody (mouse anti-SPARC (sc-73472, Santa Cruz Biotechnology, Santa Cruz, CA, USA), rabbit anti-VWF (A0082, Dako, Glostrup, Denmark), mouse anti-calmodulin (# 05-173, Merck Millipore, Darmstadt, Germany) or mouse anti-Sema4D (clone30/CD100, BD Biosciences, San Jose CA, USA)). Membranes were washed 3 times with PBS, 0.1% Tween20 followed by incubation with secondary antibodies (IRDye® 680LT Donkey anti-Mouse IgG (H+L) or Donkey anti-Rabbit IgG (H+L) (LI-COR Biotechnology)). The membranes were scanned on an Odyssey scanner (LI-COR Biotechnology) and intensities were analyzed using Image Studio Lite (Version 4.0). For A2M immunoblotting, membranes were incubated with A2M-HRP (GAA2M-AP, Affinity Biologicals, Ancaster, ON, Canada), and after 3 washes with PBS, 0.1% Tween, the membranes were incubated with ECL substrate (Roche Diagnostics) and films were developed.

### ELISA

VWF was quantified by ELISA using the RAgmix ELISA, a mix of 4 primary murine antibodies (CLB-RAg 20, CLB RAg 35, CLB-RAg 42 and CLB RAg 56)^58^ were coated on a MaxiSorp microtiter plate in coating buffer (50 mM NaHCO_3_, pH 9.8) for 16 h at 4 °C. Samples were diluted in PBS containing 0.01 Tween20 and 1% bovine serum albumin and incubate for 2 h at 37 °C. Polyclonal peroxidase-labeled anti-VWF IgG (DAKO A/S, Glostrup, Denmark) was used as secondary antibody. Normal human plasma from a pool of 30 donors was used as standard.

SPARC, PF4 and S100A9 ELISAs were obtained from R&D Systems and were performed according to manufactures protocol.

### Data availability

The.raw MS files and search/identification files obtained with MaxQuant have been deposited in the ProteomeXchange Consortium (http://proteomecentral.proteomexchange.org/cgi/GetDataset) via the PRIDE partner repository^59^ with the dataset identifier PXD005610.

## Electronic supplementary material

Supplementary Information

Supplementary Table
