# Supplementary material for: Monitoring storage induced changes in the platelet proteome employing label free quantitative mass spectrometry
Source: Sci Rep. 2017 Sep 8;7:11045. doi: 10.1038/s41598-017-11643-w (PMC5591311; doi:10.1038/s41598-017-11643-w)
Supplement: Supplementary file 1 — Supplementary Information [file 41598_2017_11643_MOESM1_ESM.pdf]

Supplementary Data

Monitoring storage induced changes in the platelet proteome employing label free quantitative mass spectrometry

Maaike Rijkers[1], Bart L. van den Eshof[1], Pieter F. van der Meer[2], Floris P.J. van Alphen[1,3], Dirk de Korte[2,4], Frank W.G. Leebeek[5], Alexander B. Meijer[1, 3, 6], Jan Voorberg[1,7]*, A.J. Gerard Jansen[1,5]

[1] Department of Plasma Proteins, Sanquin-AMC Landsteiner Laboratory, Amsterdam, The Netherlands

[2] Department of Product and Process Development, Sanquin Blood Bank, Amsterdam, The Netherlands

[3] Department of Research Facilities, Sanquin, Amsterdam, The Netherlands

[4] Department of Blood Cell Research, Sanquin-AMC Landsteiner Laboratory, Amsterdam, The Netherlands

[5] Department of Hematology, Erasmus University Medical Centre, Rotterdam, The Netherlands

[6] Department of Pharmaceutics, Utrecht Institute for Pharmaceutical Sciences (UIPS), Utrecht University, Utrecht, The Netherlands

[7] Department of Vascular Medicine, Amsterdam Medical Centre, Amsterdam, The Netherlands

*Corresponding author: Jan Voorberg PhD, Department of Plasma Proteins, Sanquin Research, Plesmanlaan 125, 1066 CX, Amsterdam, The Netherlands. Telephone: +31(0)20-512 3120, telefax: +31(0)20-512 3310, e-mail: j.voorberg@sanquin.nl

Supplementary data S1

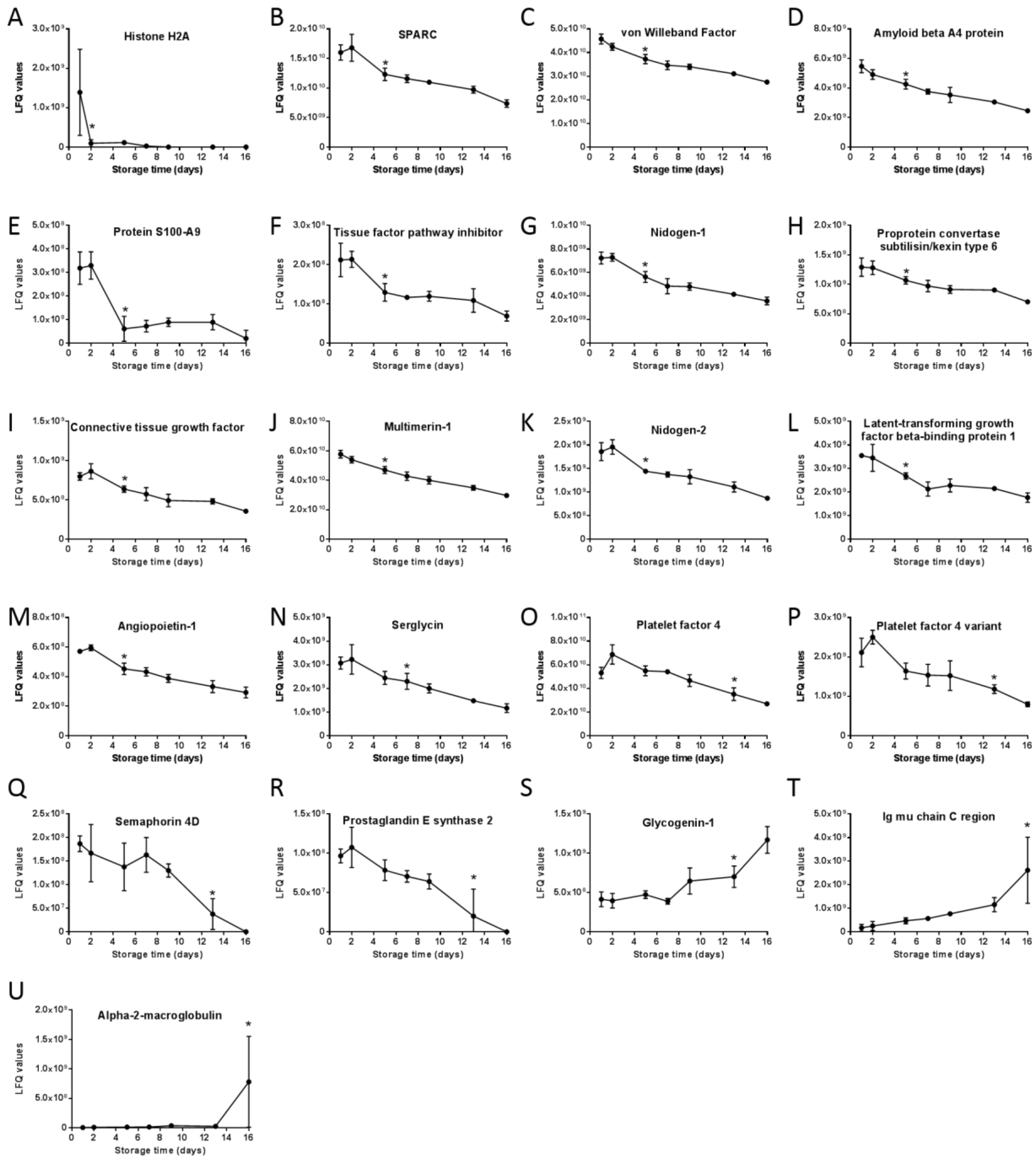

*Supplementary data S1. Non-imputed LFQ values of significantly changes proteins during platelet storage. Data represents mean ± standard deviation (n=3), *: P < 0.05 compared to day 1 from this time point onwards. LFQ values of 0 indicate that the protein was not detected at that time point.*

Supplementary data S2

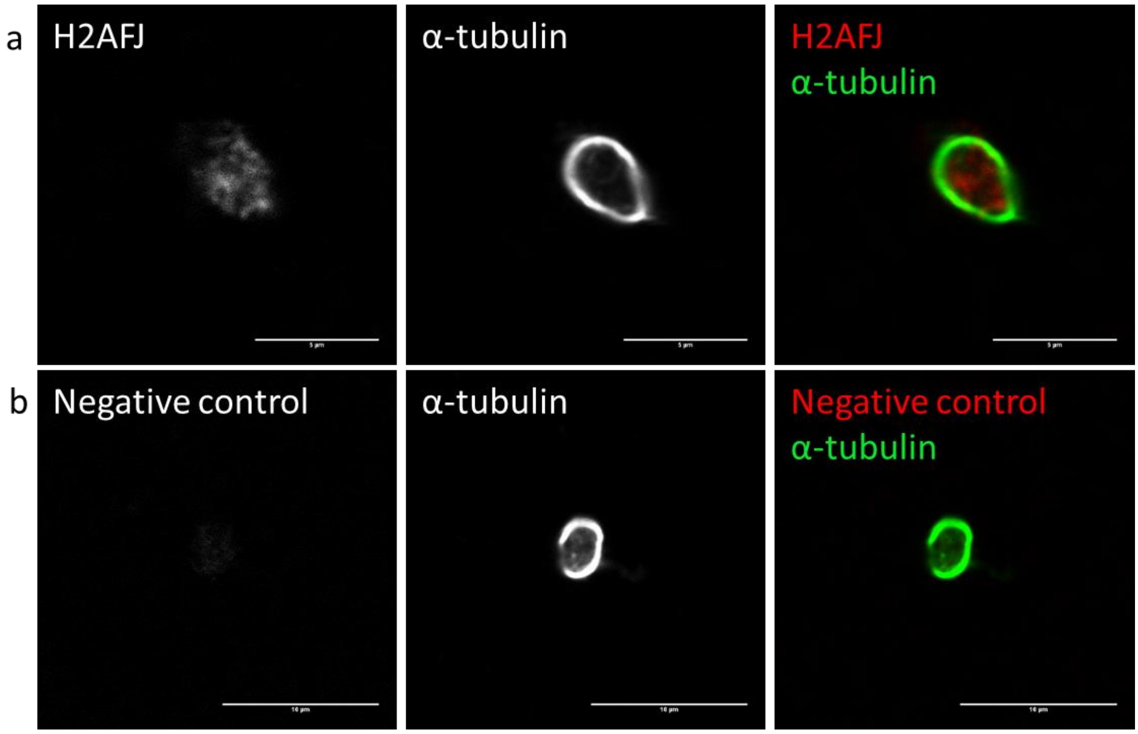

Supplementary data S2A. The peptides identified for histone H2A correspond to different histone H2A variants with highly homologous sequences (H2AFJ, HIST1H2AJ, HIST1H2AH, HIST1H2AC, HIST3H2A, HIST1H2AD, HIST1H2AG, HIST1H2AB, HIST2H2AB and H2AFX), see supplementary data S2B. A polyclonal rabbit antibody directed against a peptide within the first 30 amino acids of H2AFJ (see supplementary data S2B) was used to confirm the presence of H2A in platelets. This antibody might cross-react with other histone H2A family members. Platelets on glass coverslips were stained with (A) polyclonal rabbit anti-H2AFJ (Thermo Fisher, Rockford, IL, USA) and mouse anti-α-tubulin (Merck Millipore, Darmstadt, Germany) or (B) mouse anti- α-tubulin only. Secondary antibodies goat anti-mouse Alexa 488 and chicken anti-rabbit Alexa 647 were used for both conditions. Platelets were imaged employing a Leica SP8 confocal microscope. Images confirm the presence of H2AFJ (or another H2A variant to which the antibody might bind) in platelets. The signal was absent if platelets were only stained with the secondary antibody.

**Gene name**	**Sequence**
H2AFJ	MSGRGKQGGK VRAKAKSRSS RAGLQFPVGR VHRLLRKGNY AERVGAGAPV
HIST1H2AJ	MSGRGKQGGK ARAKAKTRSS RAGLQFPVGR VHRLLRKGNY AERVGAGAPV
HIST1H2AH	MSGRGKQGGK ARAKAKTRSS RAGLQFPVGR VHRLLRKGNY AERVGAGAPV
HIST1H2AC	MSGRGKQGGK ARAKAKSRSS RAGLQFPVGR VHRLLRKGNY AERVGAGAPV
HIST3H2A	MSGRGKQGGK ARAKAKSRSS RAGLQFPVGR VHRLLRKGNY SERVGAGAPV
HIST1H2AD	MSGRGKQGGK ARAKAKTRSS RAGLQFPVGR VHRLLRKGNY SERVGAGAPV
HIST1H2AG	MSGRGKQGGK ARAKAKTRSS RAGLQFPVGR VHRLLRKGNY AERVGAGAPV
HIST1H2AB	MSGRGKQGGK ARAKAKTRSS RAGLQFPVGR VHRLLRKGNY SERVGAGAPV
HIST2H2AB	MSGRGKQGGK ARAKAKSRSS RAGLQFPVGR VHRLLRKGNY AERVGAGAPV
H2AFX	MSGRGKTGGK ARAKAKSRSS RAGLQFPVGR VHRLLRKGHY AERVGAGAPV

H2AFJ	YLAAVLEYLT AEILELAGNA ARDNKKTRII PRHLQLAIRN DEELNKLLGK
HIST1H2AJ	YLAAVLEYLT AEILELAGNA ARDNKKTRII PRHLQLAIRN DEELNKLLGK
HIST1H2AH	YLAAVLEYLT AEILELAGNA ARDNKKTRII PRHLQLAIRN DEELNKLLGK
HIST1H2AC	YLAAVLEYLT AEILELAGNA ARDNKKTRII PRHLQLAIRN DEELNKLLGR
HIST3H2A	YLAAVLEYLT AEILELAGNA ARDNKKTRII PRHLQLAIRN DEELNKLLGR
HIST1H2AD	YLAAVLEYLT AEILELAGNA ARDNKKTRII PRHLQLAIRN DEELNKLLGK
HIST1H2AG	YLAAVLEYLT AEILELAGNA ARDNKKTRII PRHLQLAIRN DEELNKLLGK
HIST1H2AB	YLAAVLEYLT AEILELAGNA ARDNKKTRII PRHLQLAIRN DEELNKLLGR
HIST2H2AB	YLAAVLEYLT AEILELAGNA ARDNKKTRII PRHLQLAVRN DEELNKLLGG
H2AFX	YLAAVLEYLT AEILELAGNA ARDNKKTRII PRHLQLAIRN DEELNKLLGG

H2AFJ	VTIAQGGVLP NIQAVLLPKK TESQKTKSK
HIST1H2AJ	VTIAQGGVLP NIQAVLLPKK TESHHKTK
HIST1H2AH	VTIAQGGVLP NIQAVLLPKK TESHHKAK
HIST1H2AC	VTIAQGGVLP NIQAVLLPKK TESHHKAKGK
HIST3H2A	VTIAQGGVLP NIQAVLLPKK TESHHKAKGK
HIST1H2AD	VTIAQGGVLP NIQAVLLPKK TESHHKAKGK
HIST1H2AG	VTIAQGGVLP NIQAVLLPKK TESHHKAKGK
HIST1H2AB	VTIAQGGVLP NIQAVLLPKK TESHHKAKGK
HIST2H2AB	VTIAQGGVLP NIQAVLLPKK TESHKPGKNK
H2AFX	VTIAQGGVLP NIQAVLLPKK TSATVGPKAP SGGKKATQAS QEY

Supplementary data S2B. The 10 H2A variants with the corresponding sequences. In grey the homologues amino acids are depicted. In red the peptides identified by the mass spectrometry analysis have been indicated. The black box represents the first 30 amino acids of H2AFJ, to which the antibody used for the confocal microscopy analysis is directed.

Supplementary data S3

In order to confirm the platelet origin of the proteins of which levels were changed during storage, we compared our data to published datasets.

Rieckmann et al published a dataset including copy numbers of proteins identified in different primary human hematopoietic cell populations.[1] The top 20 most abundant proteins in neutrophils, monocytes, NK cells, T cells and B cells were compared to the proteins we identified to change in abundancy during storage. In neutrophils and monocytes, but not in other cell types, S100A9 was reported to be one of the most abundant proteins (based on copy number).

Contamination of leukocytes is a general problem in platelet proteomics, Zeiler et al distinguished the true murine platelet proteome from the contaminants by employing successive stages of purification.[2] By following the abundancy of proteins during these purification steps, contaminants were identified. In agreement with the paper of Rieckmann et al. S100A9 was identified as a potential contaminant.

The presence of S100A9 in platelets has been reported by mass spectrometry studies[3], RNA-seq of platelets[4] and megakaryocytes[5]. Also functional studies on platelet derived S100A9 can be found in literature.[6,7] As platelet concentrates, after filtering with a leukocyte depletion filter contain only a maximum of 10,000 leukocytes, from which 10% is monocyte or granulocyte, we assume that S100A9 we identified in our study originates from platelets. In Supplementary data 4 we provide experimental proof that S100A9 is a bona vide component of platelets.

In the study of Zeiler et al, different histones were identified as contaminants. However, the histone identified in our current study, H2AFJ is identified as a true platelet protein in the dataset of Zeiler et al. Additionally, H2AJ has been detected in platelet microparticles.[8]

As indicated in the Legend of Figure 1 peptides identified and quantified for H2A can be derived from multiple histone H2A variant. These variants include: H2AFJ, HIST1H2AJ, HIST1H2AH, HIST1H2AC, HIST3H2A, HIST1H2AD, HIST1H2AG, HIST1H2AB, HIST2H2AB and H2AFX. This issue is discussed in more detail in Supplementary data S4.

Besides H2AFJ we identified more histones, of which some have not been described to be present in platelets. Since no significant differences in levels of these histones were observed we did not include these data in the manuscript.

References

1. Rieckmann, J. C. *et al.* Social network architecture of human immune cells unveiled by quantitative proteomics. *Nat. Immunol.* **18,** (2017).
2. Zeiler, M., Moser, M. & Mann, M. Copy Number Analysis of the Murine Platelet Proteome Spanning the Complete Abundance Range. *Mol. Cell. Proteomics* **13,** 3435–3445 (2014).
3. Burkhart, J. M. *et al.* The first comprehensive and quantitative analysis of human platelet protein composition allows the comparative analysis of structural and functional pathways. *Blood* **120,** e73–e82 (2012).
4. Rowley, J. W. *et al.* Genome wide RNA-seq analysis of human and mouse platelet transcriptomes. *Blood* **118,** e101–11 (2011).

5. Meinders, M. *et al.* Sp1 / Sp3 transcription factors regulate hallmarks of megakaryocyte maturation , and platelet. *Blood* **125,** 1957–1967 (2015).

6. Lood, C. *et al.* Platelet-derived S100A8/A9 and cardiovascular disease in systemic erythematosus. *Arthritis Rheumatol.* **68,** 1970–1980 (2016).

7. Wang, Y. *et al.* Platelet-derived S100 family member myeloidrelated protein-14 regulates thrombosis. *J. Clin. Invest.* **124,** 2160–2171 (2014).

8. Garcia, B. A. *et al.* The platelet microparticle proteome. *J. Proteome Res.* **4,** 1516–21 (2005).

Supplementary data S4

The efficiency of the use of a leukocyte reduction filter was tested by taking samples from a platelet concentrate (PC), before applying filtration, after one filtration step and after 2 filtration steps. Samples were analyzed employing LeucoCOUNT (CellQuestTM Pro software). Before filtering, 217.82 x 106 / L leukocytes were present in the PC. After 1 filtration step, only 2 events could be measured, and after an additional filtration step 1 event was measured (Table 1). These results indicate that the use of a leukocyte reduction filter very efficiently removes leukocytes from platelet concentrates.

Table 1. Leukocyte counts in PCs obtained with LeucoCOUNT (CellQuest^{TM} Pro software), before and after applying a leukocyte reduction filter.

	**Before filtering**	**1 x filtering**	**2x filtering**
**Leukocyte count (events measured)**	4505	2	1
**Leukocyte count (concentration)**	2.2 x 10^8 / L	0.1 x 10^6 / L	0.05 x 10^6 / L
**Platelet count (concentration)**	1.2 x 10^{12} / L	1.2x 10^{12} / L	1.2 x 10^{12} / L

To study the efficiency of platelet washing and purification for lysate preparation and to confirm that leukocyte contamination did not affect our results, lysates of the samples before and after 1 and 2 filtration steps were prepared. To do this, the samples were spun down 20 min at 120 g. On the PRP 2 more washing steps were performed before the platelets were lysed in lysis buffer. Levels of S100A9, platelet factor 4 (PF4) and von Willebrand Factor (VWF) were measure employing ELISA (Fig. S3). Levels of S100A9, PF4 and VWF were similar before and after filtration. As S100A9 is a highly abundant protein in leukocytes, these results indicate that the method used excludes leukocytes which were at first present in the unfiltered sample. This suggests that the platelet lysates which were used for the mass spectrometry analysis (which were obtained from PCs that were filtered once with a leukocyte reduction filter) contained little to no contamination of leukocytes. Based on these data S100A9 appears to be a bona fide component of platelets and its decline during storage suggest that it may be a useful marker for monitoring of development of the platelet storage lesion.

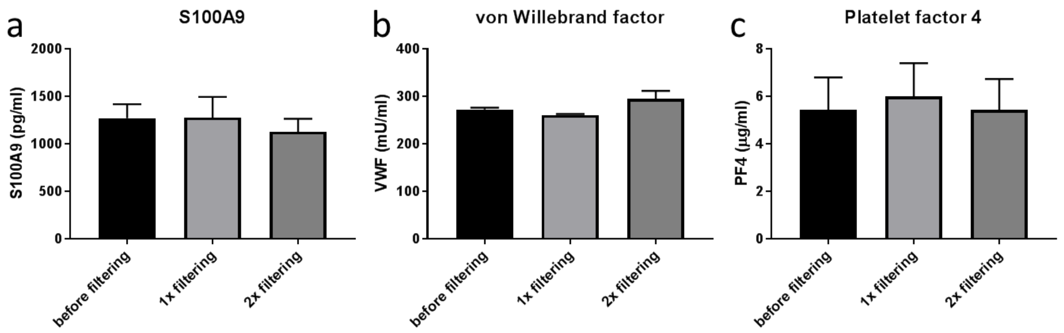

Supplementary figure S4. S100A9, VWF and PF4 levels are unchanged in platelet lysates before and after 1 or 2 filtration steps with a leukocyte reduction filter. (A) S100A9. (B) VWF. (C) PF4.

Supplementary data S5

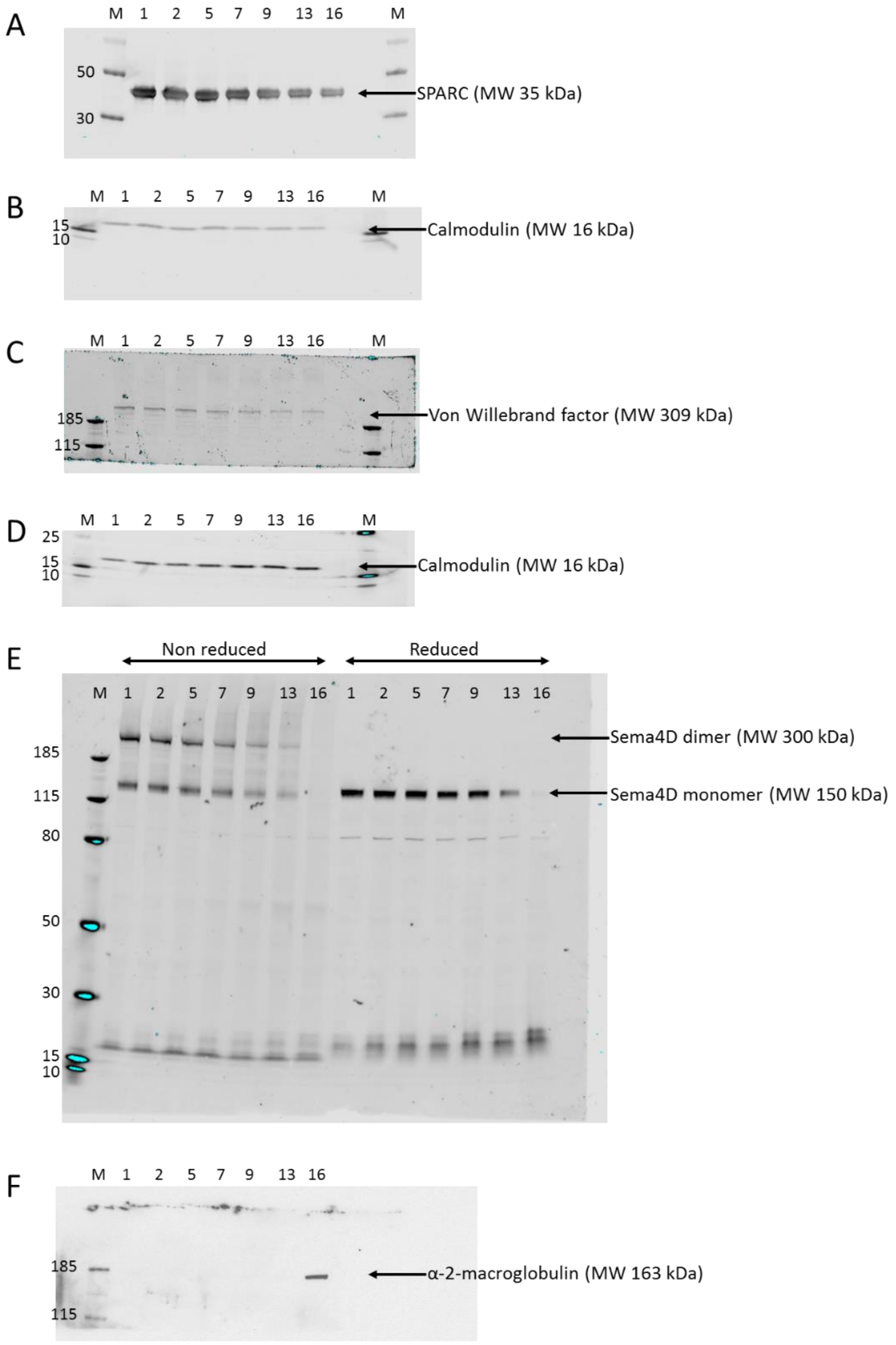

Supplementary data S5. Uncropped immunoblots. (A) SPARC (Fig 4C). (B) Calmodulin (Fig 4C and 6D). (C) von Willebrand factor (Fig 4D). (D) Fig 4D). (E) Semaphorin 4D (Sema4D) (Fig 6C). (F) Alpha-2-macroglobulin (Fig 6D). "M" indicates the molecular weight marker, number above the membrane the storage days. Molecular weight of the marker bands is indicated at the left of the immunoblots.
